# Detection of HPV related oropharyngeal cancer in oral rinse specimens

**DOI:** 10.18632/oncotarget.22682

**Published:** 2017-11-25

**Authors:** Matthew Rosenthal, Bin Huang, Nora Katabi, Jocelyn Migliacci, Robert Bryant, Samuel Kaplan, Timothy Blackwell, Snehal Patel, Liying Yang, Zhiheng Pei, Yi-Wei Tang, Ian Ganly

**Affiliations:** ^1^ Head and Neck Service, Department of Surgery, Memorial Sloan Kettering Cancer Center, New York, NY, USA; ^2^ Clinical Microbiology Service, Department of Laboratory Medicine, Memorial Sloan Kettering Cancer Center, New York, NY, USA; ^3^ Department of Pathology, Memorial Sloan Kettering Cancer Center, New York, NY, USA; ^4^ Department of Medicine, New York University School of Medicine, New York, NY, USA; ^5^ Department of Pathology, New York University School of Medicine, New York, NY, USA; ^6^ Department of Veterans Affairs New York Harbor Healthcare System, New York, NY, USA; ^7^ Department of Laboratory Medicine, First Affiliated Hospital of Sun Yat-sen University, Guangzhou, China; ^8^ Department of Pathology and Laboratory Medicine, Weill Medical College of Cornell University, New York, NY, USA

**Keywords:** human papillomavirus, oral rinse, oropharynx cancer, oral cavity cancer, screening test

## Abstract

**Background:**

The majority of patients diagnosed with oropharyngeal squamous cell cancer (OPSCC) are due to HPV infection. At present, there are no reliable tests for screening HPV in patients with OPSCC. The objective of this study was to assess the Cobas® HPV Test on oral rinse specimens as an early, non-invasive tool for HPV-related OPSCC.

**Methods:**

Oral rinse specimens were collected from 187 patients (45 with OPSCC, 61 with oral cavity SCC (OCSCC) and 81 control patients who had benign or malignant thyroid nodules) treated at MSKCC. The Cobas® HPV Test was used to detect 14 high-risk HPV types in these samples. Performance of the HPV Test was correlated with p16 tumor immunohistochemistry as gold standard.

**Results:**

91.1% of the oropharynx cancer patients had p16 positive tumors compared to 3.3% of oral cavity cancer. Of the 81 control patients, 79 (97.5%) had no HPV in their oral rinse giving a specificity of the HPV test of 98%. For the combined oral cavity oropharynx cancer cohort, the sensitivity, specificity, positive predictive value and negative predictive value of the HPV Test were 79.1%, 90.5%, 85.0% and 86.4% respectively when p16 immunohistochemistry was used as the reference.

**Conclusion:**

The Cobas® HPV Test on oral rinse is a highly specific and potentially sensitive test for oropharyngeal cancer and may be a potentially useful screening test for early oropharyngeal cancer.

**Impact:**

We describe an oral rinse test for the detection of HPV related oropharyngeal cancer.

## INTRODUCTION

The link between human papillomavirus (HPV) and oropharyngeal squamous cell carcinoma (OPSCC) has been well-documented over the past two decades [1–4]. Currently, HPV-related OPSCC comprise the majority of the new cases of the disease in the United States and the incidence rates are on the rise [5]. The prevalence of high-risk oral HPV (HR-HPV) has been reported at 3.7% of the US population with a bimodal age distribution of incidence [6]. It remains unclear why certain people go on to develop OPSCC while others clear the initial HPV infection [7]. A recent study suggests that persistent infection may be due to elevated levels of circulating inflammatory cytokines [8]. Understanding the balance of inflammatory mediators that lead to HPV carcinogenesis may help to develop a future screening test for OPSCC, but a serological test of this nature appears out of reach at this time.

Expectations are that the rate of HPV oropharynx cancer will increase in incidence over the next decade. The efficacy of current HPV vaccination practices at reducing the disease burden of oral HPV remains to be established but the rates of HPV-related OPSCC are eventually predicted to decline mirroring the decline of cervical cancer [5]. Until this decline occurs there is therefore the need for new screening and prevention methods to be established. Parallel to vaccination are the secondary prevention tools for early detection of OPSCC in its early stages. Precancerous lesions are difficult to assess within the oropharynx, so biomarker screening provides the greatest opportunity for early detection. Though seropositivity for HPV16 E6 protein may be a marker for cancer, it is unclear whether a blood test will have the desired sensitivity of an ideal screening test [9]. Since precancerous or cancerous lesions of the oropharynx may eventually be expectorated into saliva, an oral rinse test is a promising screening tool. Oral rinse is also one of the least invasive body fluids available for biomarker study and has become a more popular diagnostic tool in recent decades [10]. In this study, we sought to explore whether the oral rinse can be used as a non-invasive specimen type as a quick detection method for HPV-related OPSCC.

Previous studies in Europe have suggested that an oral rinse test might be highly specific [11, 12] and we sought to expand this work to the US with a three-cohort study involving newly diagnosed OPSCC, newly diagnosed oral cavity squamous cell carcinoma (OCSCC), and a normal control population. The addition of a normal cohort allowed us to examine the rate of false positives for the oral rinse test in screening for OPSCC. Additionally, many studies used PCR-based oral rinse techniques for detection of oral HPV in high-risk individuals [13, 14]. By comparing PCR results to the current standard used for HPV detection in cancer tissue, immunohistochemistry stain for p16, we sought to further evaluate the performance of the oral rinse technique.

## RESULTS

### Patient demographics and clinical characteristics

Our study included 187 patients separated into three cohorts based on tumor site: there were 45 patients with OPSCC tumors, 61 with OCSCC tumors, and 81 patients in our normal cohort. Table 1 shows a demographic summary of these patients. We carried out a chi-square analysis to examine the differences between the two tumor cohorts. As expected, there was a significant difference between the two groups for tumor p16 positivity; 91.1% of the oropharynx cancer patients were p16 positive compared to 3.3% of oral cavity cancer patients (p<0.001). This distribution reflects the different etiologies of oropharyngeal and oral cavity cancers with oropharynx cancers largely caused by HPV whereas oral cavity cancer largely caused by smoking and alcohol. This difference is also reflected in the clinical and pathologic regional lymph node (N) stages of the two group (p<0.001 and p<0.001 respectively). A large majority of the patients included in the oral cavity cohort presented with clinically negative necks (78.7%) and also had negative necks on pathology (70.0%). In contrast, the majority of the oropharynx patients presented at their initial consultations with clinical nodal metastases (97.8%). It should be noted that there was limited pathologic T stage or N stage for the oropharynx cohort because the majority of these patients were treated with chemoradiation and did not have surgical resection done (11 of the 45 had surgery).

**Table 1:** Demographic data and clinical characteristics for each cohort

Variable		Oropharynx (n=45) %	Oral (n=61) %	p-value (chi Sqr)	Normal (n=81) %
**Age**	≤60	24 (53.3%)	24 (39.3%)	0.153	64 (79.0%)
	>60	21 (46.7%)	37 (60.7%)		16 (19.8%)
**Gender**	Male	38 (84.4%)	30 (49.2%)	**<0.001**	15 (18.5%)
	Female	7 (15.6%)	31 (50.8%)		66 (81.5%)
**Smoking**	Never	14 (31.1%)	26 (42.6%)	0.227	35 (43.2%)
	Ever	31 (68.9%)	35 (57.4%)		39 (48.1%)
**Alcohol**	Never	7 (15.6%)	15 (24.6%)	0.257	11 (13.6%)
	Ever	38 (84.4%)	46 (75.4%)		62 (76.5%)
**Clinical T Stage**	T1	5 (11.1%)	27 (45.8%)	NA	
	T2	30 (66.7%)	19 (32.2%)		
	T3	5 (11.1%)	4 (6.8%)		
	T4	5 (11.1%)	9 (15.3%)		
**Clinical N Stage**	N0	1 (2.2%)	48 (78.7%)	**<0.001**	
	N+	44 (97.8%)	13 (21.3%)		
**Clinical M Stage**	M0	44 (97.8%)	61 (100%)	0.425^*^	
	M1	1 (2.2%)	0		
**Overall Clinical Stage**	I	0	25 (42.4%)	**<0.001**	
	II	1 (2.2%)	14 (23.7%)		
	III	5 (11.1%)	8 (13.6%)		
	IV	39 (86.7%)	12 (20.3%)		
**Pathologic T Stage (n=47)**	T1	4 (36.4%)	33 (56.9%)	NA	
	T2	7 (63.6%)	15 (25.9%)		
	T3	0	2 (3.4%)		
	T4	0	8 (13.8%)		
**Pathologic N Stage (n=48)**	N0/NX	1 (9.1%)	42 (70.0%)	**<0.001**	
	N+	10 (90.9%)	18 (30.0%)		
**Overall Pathologic Stage**	I	0	28 (48.3%)	NA	
	II	1 (9.1%)	9 (15.5%)		
	III	2 (18.2%)	4 (6.9%)		
	IV	8 (72.7%)	17 (29.3%)		
**p16 Tissue**	negative	4 (8.9%)	59 (96.7%)	**<0.001**	
	positive	41 (91.1%)	2 (3.3%)		
**HPV Oral rinse**	HPV 16 positive	33 (73.3%) ^*^	6 (9.8%)	NA	2 (2.5%)
	HPV 18 positive	1 (2.2%)	0		0 (0.0%)
	HR-other HPV positive alone	2 (4.4%)	2 (3.3%)		1 (1.2%)
	negative	9 (20.0%)	53 (86.9%)		78 (96.3%)

Comparison of the oropharynx and oral cavity cohorts was carried out by the Chi-square test of association.

^*^18 of 33 were also positive for another HPV serotype.

### Prevalence of HPV in oral rinse of normal, oral cavity cancer and oropharynx cohorts

Our normal cohort consisted of 81 patients who attended the head and neck clinic with either thyroid cancer or benign thyroid nodules. All patients had a comprehensive head and neck examination. This involved an examination of the oral cavity and oropharynx and either flexible or mirror laryngoscopy to examine the larynx, hypopharynx and base of tongue. All control patients had no clinical evidence for oral cavity cancer or oropharyngeal cancer. Of the 81 patients, 79 (97.5%) had no HPV in their oral rinse on PCR. Two patients were positive for HPV 16 but clinical examination showed no clinical evidence for tonsil, base of tongue cancer or oral cavity cancer. These results indicate the very high specificity (97.5%) of the Cobas® HPV Test for the detection of HPV with only 2 false positive results from 81 control patients studied.

Our oral cavity cancer cohort consisted of 61 patients with the majority of patients with cancer of the oral tongue (n=40). 6 patients (9.8%) had a positive HPV oral rinse test and this was for HPV16. Of these 6 patients, 2 had oral tongue, 1 lower gum, 1 floor of mouth, 1 retromolar trigone and 1 buccal mucosa cancer. Two of the 6 patients with a positive oral rinse test were confirmed to have p16 positive tumors (1 oral tongue cancer and 1 retromolar trigone). The other 4 patients who had a positive HPV oral rinse test had negative p16 immunohistochemistry in the oral cavity tumors; these 4 patients are currently under follow up but none have developed an oropharyngeal cancer to date.

The oropharynx cancer cohort consisted of 45 patients. Of these, 34 of 45 (76%) patients were positive for HPV16 or 18 in their oral rinse (33 HPV16 and 1 HPV18). Of the 34 patients, 32 had p16 positive tumors and 2 patients had p16 negative tumors. Of the 33 pts that were HPV16 positive in their oral rinse, 18 were also positive for another HPV virus serotype. In addition, of the 11 patients that were negative for HPV16/18 on oral rinse, 2 were positive for another HPV serotype.

### Sensitivity, specificity, positive predictive value, and negative predictive value of HPV oral rinse PCR test for detection of HPV positive head and neck cancer

Table 2 shows the sensitivity, specificity, positive predictive value and negative predictive value of Cobas®HPV Test for detection of HPV (p16) positive head and neck cancer. There were 43 patients with p16 positive tumors (41 patients with OPSCC and 2 patients with OCSCC both of whom had oral tongue SCC). Of these, the HPV Test was positive on oral rinse in 34 patients giving a sensitivity of 79.1% (Confidence interval 0.64, 0.90). There were 63 patients with p16 negative tumors (4 patients with OPSCC and 59 patients with OCSCC). Of these, the HPV Test was negative in 57 patients giving a specificity of 90.5% (Confidence interval 0.80, 0.96).. The positive predictive value of the HPV Test was 85.0%(Confidence interval 0.70, 0.94) and the negative predictive value 86.4% (Confidence interval 0.76, 0.94).

**Table 2:** Comparison of oral rinse results with tumor status: both oropharynx and oral cavity cohorts combined

		Tumor p16 Status		Total
		Positive	Negative	
**Oral rinse Results by HPV PCR**	**HPV16 or HPV18 Positive**	34	6	40
	**HPV16 or HPV18 Negative**	9	57	66
Total		43	63	106

Sensitivity = 79.1%, Specificity =90.5%, PPV = 85.0%, and NPV = 86.4%.

We also present these data with the oral cavity cohort removed, and then broken down into oropharynx subsite for comparison (Table 3). The positive predictive value of the Cobas® HPV Test for oropharynx cancers was 94% with a sensitivity of 78%. When further divided by subsite, the test has a 100% positive predictive value for OPSCC of the tonsil, while a slightly diminished value for the base of tongue (86.7%).

**Table 3:** Comparison of oral rinse results with tumor status: total oropharynx and subsite-specific results shown

Total oropharynx		Tumor p16 status		Total
		Positive	Negative	
**Oral rinse Results of HPV PCR**	**HPV16 or HPV18 Positive**	32	2	34
	**HPV16 or HPV18 Negative**	9	2	11
Total		41	4	45
Sensitivity = 78%, Specificity = 50%, PPV = 94%, and NPV = 18%.				

Tonsil		Tumor p16 status		Total
		Positive	Negative	
**Oral rinse Results of HPV PCR**	**HPV16 or HPV18 Positive**	19	0	19
	**HPV16 or HPV18 Negative**	3	0	3
Total		22	0	22
Sensitivity = 86%, Specificity = not evaluable, PPV = 100%, and NPV = not evaluable.				

Base of tongue		Tumor p16 status		Total
		Positive	Negative	
**Oral rinse Results of HPV PCR**	**HPV16 or HPV18 Positive**	13	2	15
	**HPV16 or HPV18 Negative**	6	2	8
Total		19	4	23
Sensitivity = 68%, Specificity = 50%, PPV = 87%, and NPV = 25%.				

## DISCUSSION

The patients selected for our oropharynx and oral cavity cohorts were not chosen based on any known information regarding their HPV status. However, p16 testing confirmed that the OPSCC population was 91.1% (41/45) positive for HPV, while our OCSCC population was only 3.3% (2/61) for HPV. Analysis showed these groups to be significantly different in the p16 status (p<0.001) which is consistent with the observed demographic differences in these two diseases over the past 2 decades, where HPV has become a predominant cause of OPSCC [15]. Additionally, there was a statistically significant difference in their gender (p=0.014), where the OPSCC group showed a greater male predominance (84.4% versus 60.5% of OCSCC), and a higher N stage at presentation (p<0.001). These differences are reflective of demographic trends for these two diseases. Recent studies have shown that HPV-positive OPSCC patients are more likely to be younger, male, and to present with nodal metastasis [16]. There were more OPSCC patients under the age of 60, but the differences between the age of the two groups was not statistically significant (p=0.208). This may have been an artifact of the relatively small sample size in our study.

The purpose of this project was to evaluate the potential for an oral rinse test as an early detection tool in head and neck squamous cell carcinoma. Our analysis proved this test to be fairly sensitive (79.1%) and highly specific (90.5%) with a PPV of 85.0% and NPV of 86.4%. This suggests that the Cobas® HPV Test has value in characterizing lesions of the oropharynx or oral cavity. The test was particularly sensitive to HPV-related cancers of the tonsil (86.4%) with a PPV of 100%. However, the results were less consistent for tumors of the base of tongue (68.4% sensitivity, 50% specificity). The fact that these HPV markers were more likely to be detected in tonsil cancer than base of tongue cancer may be a result of the anatomy of the oropharynx. Portions of the tonsils are more likely to have contact with an oral rinse collection when a patient is asked to gargle and expectorate saline solution as in this study. Additionally, the number of viral copies of HPV has been shown to be greater in cancers of the tonsil than other sites [17]. Previous studies have also established that tonsil cancers are more likely to be positive for HPV on an oral rinse test than oral cavity cancer [18], and indeed our results confirmed this. The observation that the oral rinse test is less sensitive in the detection of HPV related base of tonue cancers is a limitation of the test but since tonsil cancers are more common than base of tongue cancers the oral rinse test will identify a large proportion of HPV related oropharyngeal cancers. Two patients (2.5%) in our normal cohort were shown to be positive on oral rinse for HPV16. This is comparable to published data that suggests that 0.7% of healthy individuals in developed nations are HPV16 positive [19].

This study and others like it [11, 12], suggest a role for the oral rinse test in screening for OPSCC. Unlike other studies, our study used a large HPV negative control cohort, which allowed us to determine the false positive rate and specificity of the Cobas® HPV Test. In addition, our study was larger overall compared to previous studies. Yoshida et al. recently published a study with a similar three cohort distribution to ours. However, they were limited by a small number of HPV positive test results. In addition, their HPV negative cohort consisted of samples from many different sites in the head and neck [20]. Our study has larger overall groups, a more standardized HPV negative control cohort and therefore our conclusions are more robust. Whether or not our test can be applied to the general population is debatable. The detection of HPV virus in 2.5% of control patients who do not have oropharyngeal cancer would mean the investigation of many patients who do not have oropharyngeal cancer. This may not be a cost effective screening strategy. Limiting screening with the oral rinse sample to a high risk group, such as males over 50 years of age, who attend their primary care physician or dentist, may be a more cost effective strategy.

A recent study has also suggested that the oral rinse test could be used as a post treatment prognostic indicator [21]. However these conclusions relied on a small number of positive test results and therefore larger studies are required to validate these findings. Our study suggests the oral rinse test may have greater potential in a pretreatment environment. The Cobas® HPV Test is a commercially available noninvasive tool, inexpensive and quick. Since rates of HPV-related OPSCC are on the rise [22], it is important to develop tools such as this to help diagnose these patients early with smaller primary tumors. The oral rinse test could identify patients at an earlier primary tumor stage (T1 tumors less than 2cm and T2 tumors 2-4cm in size) and this could have important implications on cost of care. For example, patients who present with smaller cancers and who have small volume neck disease can now be treated with less intensive treatment. This treatment includes either radiation alone, surgery alone or surgery combined with radiation. In contrast, patients with advanced primary tumors (Tumors staged as T3 or T4) with advanced neck disease are treated with combined modality therapy with chemoradiation. The cost of care with chemoradiation is substantially more expensive than radiation alone, surgery alone or surgery with radiation. Thus the oral rinse test could potentially result in reduced cost of care by identifying patients more suitable for these less intensive treatments.

Our study has several limitations. Although we had larger study cohorts than other studies of the oral rinse test, larger studies are still required to further validate these findings. An expansion of this study to a greater population through multi-institutional collaboration is warranted. In this study, we used an off-label process to extract nucleic acids before the nucleic acid amplification step run on the cobas z 480, which may have contributed to reduced sensitivity. Although HPV positivity confers a more favorable prognosis for head and neck cancer [23] there is still great value in early detection of these tumors. Studies indicate that advanced T stage (T3T4 tumors) is an important prognostic factor in HPV positive tumors, while nodal stage is less predictive of outcome [24, 25, 26]. An oral rinse-based screening test has the potential to find more of these tumors at an earlier T stage (T1T2 tumors).This could result in de intensification of treatment using single modality treatments with either radiation or surgery or surgery combined with postoperative radiation. This less intense treatment has substantial healthcare cost reductions compared to chemoradiation which is the current treatment modality used in the majority of these cancers. With further research, these methods may be applicable in either a primary care or dental office setting.

## MATERIALS AND METHODS

### Study population

We collected oral rinse specimens from patients presenting to the Head and Neck service at Memorial Sloan Kettering Cancer Center (MSKCC). This study was approved by the MSKCC Institutional Research Board (IRB 15-256) and patients gave informed consent to use their oral rinse specimens and tumors for HPV analysis. Study participants were selected from three separate cohorts: (1) patients with biopsy proven OPSCC, (2) patients with biopsy proven SCC of the oral cavity (OCSCC) and (3) a non-SCC cohort of patients comprised of patients with benign or malignant thyroid nodules. The patients from our third cohort were deemed to be a representative “normal” population because complete head and neck examination including flexible laryngoscopy or mirror laryngoscopy of the laryngopharynx did not show any evidence for oral cavity or oropharyngeal pathology.

Patients were instructed to swish and gargle 10 mL of 0.9% NaCl solution for 30 seconds before expectorating into a sterile 50 mL vial. The vials were then stored on ice for transport to our institution’s microbiology lab.

### HPV DNA detection in oral rinse

Mouthwash samples were initially processed by spinning for 20 minutes at 2916 x *g* and 4°C on a Sorvall Legend RT. The supernatant was then separated from the pellet and both samples were stored at -80°C. DNA was extracted from the pellet using the PowerLyzer PowerSoil DNA Isolation Kit (MoBio, Carlsbad, CA, USA) per the manufacturer’s guidelines [27]. Extracted DNA samples were then analyzed on the cobas z480 (Roche Diagnostics, Pleasanton, CA, USA). The instrument performed real-time polymerase chain reaction (qPCR) on a 200bp sequence from the HPV L1 region, targeting 14 “high-risk” genotypes, while providing genotyping data on HPV 16 and 18 [28].

Cobas® HPV Test (Roche Diagnostics, Indianapolis, IN), which was approved by the US FDA for diagnostic and screening use in cervical specimens, allows HPV16 and 18 genotyping concurrently with the detection of 12 other high-risk HPV types (HPV31, 33, 35, 39, 45, 51, 52, 56, 58, 59, 66 and 68). The test reports the genotypes of the other 12 high risk HPV types as “other”. The HPV Test performs PCR amplification and real-time detection in an automated fashion (the oche cobas c4800 System). Detection of the human β-globin gene is used as an internal control to monitor specimen cellularity [29]. In this study, we adapted the Cobas® HPV Test for use in oral rinse samples using the Cobas® HPV Test module on cobas z 480 according to the manufacturers’ instructions [29].

### HPV status in OPSCC and OCSCC tumor samples by p16 immunohistochemistry

The gold standard test for the detection of HPV in tumor specimens is an invasive test by carrying out a biopsy of the tumor and then detecting HPV DNA or RNA in the specimen by *in situ* hybridization or by PCR. Ang et al. has reported that the expression of p16INK4a by immunohistochemistry correlated well (kappa = 0.80; 95% CI, 0.73 to 0.87) with the presence of HPV DNA in tumors [26]. This is cheaper and easier to carry out than ISH and PCR and therefore immunostaining of tumor sections for p16INK4a is now used as an indirect marker for HPV status in clinical pathology laboratories around the world [30]. In prospective randomized trials on treatment of patients with HPV related oropharyngeal cancer, p16 immunohistochemistry is now used as the surrogate marker for HPV positivity in the USA. However, rarely some p16 positive tumors may not be HPV related. The addition of HPV PCR to the detection methodology would increase specificity as described by Prigge et al. [31] but unfortunately DNA was not available from tumor samples in our study.

In our study, all pathology specimens were examined by a single pathologist specialized in head and neck pathology (NK) for p16 status. p16 immunohistochemistry was performed as follows: Four-micrometer tumor sections were deparaffinized, and after heat-induced epitope retrieval, immunohistochemistry for p16INK4a was performed with the primary antibody dilution of 1:7 as per manufacturer’s protocol (CINtec Histology Kit, catalog #9517, Roche mtm Laboratories AG, Heidelberg, Germany). Cases with nuclear and cytoplasmic immunolabeling in at least 70% of the tumor cells were considered positive for p16.

### Statistical analysis

Statistical analysis was carried out using SPSS (ver21, IBM Corporation, Armonk, NY). Pearson’s chi-squared test was used to compare variables between groups. Confidence intervals were calculated with the R package epiR as described by Collett (1999) [32].
